# The transferability of microsatellite loci from a homoploid to a polyploid hybrid complex: an example from fine-leaved *Festuca* species (*Poaceae*)

**DOI:** 10.7717/peerj.9227

**Published:** 2020-06-01

**Authors:** Przemysław P. Tomczyk, Marcin Kiedrzyński, Iwona Jedrzejczyk, Monika Rewers, Pawel Wasowicz

**Affiliations:** 1 Department of Geobotany and Plant Ecology, Faculty of Biology and Environmental Protection, University of Lodz, Lodz, Poland; 2 Laboratory of Molecular Biology and Cytometry, Department of Agricultural Biotechnology, UTP University of Science and Technology, Bydgoszcz, Poland; 3 Icelandic Institute of Natural History, Akureyri, Iceland

**Keywords:** Population genetics, Loliinae, Pooideae, SSR, Endemic species, Molecular ecology, Genetic diversity, Grasses, Polyploidy

## Abstract

**Background:**

Microsatellite loci, or single sequence repeats (SSR), are widely used as powerful markers in population genetics. They represent an attractive tool for studying plants such as grasses, whose evolution is driven by hybridisation and polyploidization. However, the development of microsatellite markers has been challenging and time-consuming, especially for non-model organisms lacking available genome-wide sequence data. One straightforward and low-cost approach is to transfer the SSR loci developed for one species, or complex, to another closely-related one. This work evaluates the transferability of microsatellite loci from homoploid to allopolyploid complexes of fine-leaved *Festuca* species and to assess their use in two new species. The studied complex (*F. amethystina*—*F. tatrae*) is a useful model for research on the local adaptability of grasses with different ploidy levels. Since both species can be considered as rare or threatened (*F. tatrae*—as a mountain and narrow endemic species and *F. amethystina*—a mountain species with relict lowland populations), any tool enabling studies on genetic diversity and population genetics, such as SSR markers, could also be very useful in a conservation context.

**Methods:**

The ploidy level within populations was estimated using flow cytometry. One diploid and one tetraploid population of *F. amethystina* and a diploid population of *F. tatrae* were chosen to test the transferability of SSR loci. Because our work describes the transfer of SSR nuclear markers designed originally for *F. gautieri*, a phylogenetic tree was prepared based on the ITS marker to assess the genetic distance between the studied complexes. The PCR products were separated on a high-resolution agarose gel, intended for SSR marker analysis. Appropriate solutions for the allotetraploid population and whole mixed-ploidy complex were implemented.

**Results:**

Flow cytometry confirmed earlier data regarding DNA content in the investigated species and cytotypes. The phylogenetic ITS tree indicated a small genetic distance between *F. gautieri* complexes and the studied species. Ten microsatellite markers were successfully transferred. All markers were polymorphic. In total, 163 different alleles were scored from the 10 SSR loci. PCoA of accessions revealed well-separated groups corresponding to studied populations. Over 60% of the total variance is explained by differentiation within populations and one third among them.

**Conclusions:**

The transferred markers are valid tools for the study of population genetics and inheritance relationships within cytotypes and species and between them. The presented markers can be used to study inbreeding depression in the *Festuca* species, and variations in the degrees of genetic diversity between different cytotypes in mountain and lowland areas. Our findings can also be applied to study conservation strategies for ensuring biodiversity at the genetic level in polyploid complexes.

## Introduction

The grasses (*Poaceae)* have a great impact on the structure and function of most terrestrial ecosystems, as well as on the human economy (Gibson, 2009). Studies on their evolutionary history suggest that the grasses have recently diversified as a family (Kellogg, 2001). Although the evolution of grasses is believed to be driven primarily by hybridisation and polyploidization (Hilu, 2007), several unresolved questions have been raised over the methodology of their study (Kellogg, 2016).

The results of speciation in grasses caused by homoploid or polyploid hybridisation can be unravelled by the implementation of an integrative molecular approach examining a combination of genetic markers (Catalán, 2006). Although genomic analysis is becoming increasingly popular in novel evolutionary studies, its results remain imperfect in the case of polyploid systems (Meirmans, Liu & van Tienderen, 2018). Such circumstances require the use of methods with more thoroughly tested bases.

An attractive tool that has been frequently used for genetic analysis, including studies focused on genetic diversity (see: Varshney, Graner & Sorrells, 2005), the genetic structure of populations (see: Zhou, Xie & Ge, 2003) and on evolutionary relationships (see: Kalia et al., 2011), involves the analysis of microsatellite loci, also known as single sequence repeats (SSR). SSR markers, typically 1–10 nucleotides in length, contribute significantly to the makeup of the repetitive regions of the genome. They are considered to be highly polymorphic, being widely distributed in genomes, as well as co-dominant and highly reproducible; in addition, they are also unstable, with a mutation rate of 10^3^–10^6^ per generation (Goldstein et al., 1999; Ellegren, 2000). One of the most straightforward and low-cost approaches for their study involves the transfer of the SSR loci developed for one species, or a complex of taxa, to another closely-related one. However, as the microsatellite markers need to work with all species of the diploid-polyploid complex, which may not be possible in practice, their selection can be difficult. In addition, care should be taken to determine the ploidy level of each studied population, and appropriate analyses must be used for the mixed-ploidy complexes. Furthermore, the tools for the analyses may not yet exist.

The present study examined the transferability of microsatellite loci to a polyploid hybrid complex of fine-leaved fescue: *Festuca amethystina* L. and *F. tatrae* (Czakó) Degen. (subfamily *Pooideae* and tribe *Loliinae*). *Festuca amethystina* displays a tetraploid cytotype (4×): probably a result of simultaneous hybridization and polyploidization processes between diploids of *F. amethystina* and *F. tatrae* (Šmarda et al., 2008).

Studied species display significant differences in geographical range: *F. tatrae* is endemic to the Western Carpathians (Turis et al., 2014; Chadburn & Romand-Monnier, 2014; Mráz et al., 2016)) whereas *F. amethystina* is widely distributed in Central and South-Eastern Europe. Both species occur in mountain habitats such as subalpine grasslands and relict pine forests on limestones; however, *F. amethystina* has also been confirmed from highland and lowland oak woods (e.g., Jakubowska-Gabara, 1994; Indreica, 2007; Roleček, 2007; Kiedrzyński et al., 2015; Kiedrzyński et al., 2017).

Both species can be considered as rare or threatened: *F. tatrae* is a mountain and narrow endemic species, while *F. amethystina* is a mountain species with relict lowland populations. Therefore, any tool that enables studies on genetic diversity and population genetics, including those based on SSR markers, could also have may applications in a conservation context.

Among the fine-leaved *Festuca* species, those of economic importance, such as *Festuca rubra* or *Festuca ovina* possess the best-known microsatellite markers (Jensen, Holm & Lübberstedt, 2007). Most of the SSR markers used in fine-leaved fescues have been transferred from other grasses, typically crops (e.g., Fu et al., 2006; Armoniene et al., 2010). However, the development of SSR markers strictly useful for fescues, such as those based on *Lolium multiflorum* × *Festuca glaucescens* F_1_ hybrid intended for use in the *Festuca–Lolium* complex, is still underway (Lauvergeat et al., 2005).

Regarding the fine-leaved fescues, few studies have examined the use of SSR markers in diploid-allopolyploid complexes. Although markers have been transferred from wheat and barley to three polyploid fine-leaved fescues (Fu et al., 2006), scarce analysis was performed on the complex of parental and descendant cytotypes; the results obtained from such an analysis could enable deeper research on evolutionary processes in this group of grasses.

Unfortunately, the genetic markers which can be used to study population genetics in the *F. amethystina*—*F. tatrae* complex are unknown. The aim of the present study is to assess the transferability of microsatellite loci developed for other fine-leaved fescues to a studied allopolyploid system and to evaluate their use for population genetics in this context.

## Materials & Methods

### Plant material

Forty-eight accessions of *F. amethystina* and 24 accessions of *F. tatrae* were collected. Accessions of *F. amethystina* were obtained from (1) Garmisch-Partenkirchen, Germany (47°34′60.00″N;  11°9′0.00″E, 740 m asl) and (2) Mayrwinkl, Austria (47°44′34.08″N; 14°19′7.31″ E, 668 m asl) in 2016. Accessions of *F. tatrae* were collected in Zuberec, Slovakia (49°14′0.32″ N; 19°35′55.24″E, 835 m asl) in 2017 and 2018. Plant material was immediately dried and stored in silica gel.

### Flow cytometry

The ploidy level of the investigated plants was estimated by flow cytometry based on genome size analysis. The nuclear DNA content was measured in dry leaves of all accessions. The samples for flow cytometric analysis were prepared as described previously (Rewicz et al., 2018), using 1 ml of nucleus-isolation buffer (200 mM Tris; 4 mM MgCl_2_ ×6H_2_O; 0.5% (v/v) Triton X-100; pH 7.5; Zenkteler & Jedrzejczyk, 2012) supplemented with propidium iodide (PI 50 µg/ml) and ribonuclease A (RNase A 50 µg/ml). For each sample, the nuclear DNA content was measured in 5,000–7,000 nuclei using a CyFlow Ploidy Analyser (Sysmex Partec GmbH, Görlitz, Germany) and linear amplification. The obtained histograms were analysed by the CyFlow Cube program (Sysmex Partec GmbH). Genome size was calculated using the ratio of *Festuca/P. sativum* cv. ‘Set’ (2C = 9.11 pg; Śliwińska, Zielińska & Jedrzejczyk, 2005) 2C peak positions on the histogram of fluorescence intensities. The mean coefficient of variation (CV) of the 2C peak for *F. tatrae* was 5.72%, while for diploid *F. amethystina* was 4.58% and tetraploid *F. amethystina* reached 3.69%.

### Identifying accurate SSR markers

The study followed an established procedure for developing and identifying SSR markers (Rosetto, 2003; Selkoe & Toonen, 2006), beginning with a literature review focusing on microsatellite primers developed for taxa closely related to *F. amethystina* and *F. tatrae*. This approach is a straightforward and inexpensive method that has already been used in previous studies (Cordeiro et al., 2001; Rosetto, 2003; Saha et al., 2004; Giraldo et al., 2005; Huang et al., 2016; Mansour, Bryngelsson & Garkawa-Gustavvson, 2016; Kotrappa, Hendre & Rathinavelu, 2017).

### Assessment of genetic distance between complexes

To determine the genetic/phylogenetic distance between the studied species and the source taxa providing the nuclear SSR markers, a phylogenetic tree was prepared based on the ITS nuclear marker. This was performed using the NJ clustering method and the Maximum Composite Likelihood method to measure evolutionary distances (Tamura, Nei & Kumar, 2004) in MEGA6 software (Tamura et al., 2013). The tree was constructed using six sequences of *F. amethystina* (three for each cytotype; GenBank Accession numbers: MN783289, MN783290, MN783291, MN783292, MN783293, MN783294), two sequences for *F. tatrae* (MN783295, MN783296) and the following sequences from GenBank: T *Brachypodium distachyon*: JX665601.1; *Festuca eskia*: KP296034.1; *Festuca gautieri*: AF303414.1; *Festuca norica*: EF584955.1; *Festuca occidentalis*: EF584956.1; *Festuca ovina*: JQ972950.1; *Festuca pallens*: AY254373.1; *Festuca picoeuropeana*: KP296038.1; *Festuca pyrenaica*: AF303423.1; *Festuca valesiaca*: EF584978.1; *Festuca violacea*: EF584979.1.

### DNA extraction

DNA was isolated from silica gel-dried leaves of *F. amethystina* and *F. tatrae* using Syngen Plant DNA MINI Kit following the manufacturer’s instructions.

### PCR

Microsatellites were amplified by PCR reactions using 10 microsatellite primers described for *F. gautieri* (Segarra-Moragues & Catalán, 2011). PCR was performed according to Segarra-Moragues & Catalán (2011) with minor modifications. The total volume of the PCR mix was 20 µL: 10 µL of master mix reaction buffer (DreamTaq Green PCR Master Mix, Thermo Scientific: DreamTaq DNA polymerase (DreamTaq Green buffer, dATP, dCTP, dGTP and dTTP, 400 µM each, and 4000 µM MgCl_2_), 1.6 µL of each of the labelled (forward) and unlabelled (reverse) primers (5 µM), 4.8 µL of water (PCR quality) and 2 µL of template DNA (10 ng/µL). The PCR program consisted of one step of denaturation at 94 °C for 4 min; followed by 35 cycles of denaturation (1 min at 94  °C), annealing (1 min at relevant annealing temperature, see Table 1 for details) and extension (45 s at 72 °C). A final extension step of 7 min at 72 °C was applied (Segarra-Moragues & Catalán, 2011).

**Table 1:** Characteristics of 10 microsatellite loci originally developed for *F. gautieri* (Segarra-Moragues & Catalán, 2011) and useful for research on *F. amethystina* and *F. tatrae*. For each locus, the primer pair sequences, repeat motif, size of the original fragment (bp), annealing temperature, and GenBank accession numbers are shown, as well as modifications in relation to original protocols (Segarra-Moragues & Catalán, 2011): an asterisk (*) in the annealing temperatures column indicates a change in temperature from the original protocol was needed to provide clear PCR bands, and that a PCR enhancer was used.

Locus	Primer sequence (5′–3′) b	Repeat motif	Size	Ta (°C)	GenBank Accession No.
FgauA02	F: CGTTTCAGTGTCGTTGATGTC R: TTCTCTGCGTGGTCTGTATTG	(CA)_13_	176	56	JN040543
FgauA04	F: AAGGAAGCACACTACCTACACG R: ATCCCAATCTGAACCCAATC	(CA)_10_	294	51*	JN040544
FgauA111	F:TGACCTAAACTGTTCCCAAATG R: CATGCAAGGTTGTATCTCACG	(GT)_23_	209	51	JN040545
FgauA121	F:TGGAGAGGAACTTAGTTGAAAG R:TGTACGACATGCTGATCTACA	(CA)_13_	119	51*	JN040546
FgauB07	F:TCATCGCTGACAAACTCTTC R: CTGACGGGTATTACTTCCAAC	(CT)_16_	275	51*	JN040547
FgauB103	F:CCACCTGTCATAAGCCTTTC R: GCTGATGTCCTCTTCTCGTC	(GA)_6_G(GA)_11_	138	51	JN040548
FgauB109	F:CATGGCTTGACACTCTATGAG R: TTTCAGTAAAGGGAACATCTTG	(GA)_13_	217	56*	JN040549
FgauB119	F:GGGACACAAGCACTAAAGTTG R: CCAAAAACAAAATAGGACGAAG	(GA)_15_	146	51	JN040550
FgauB125	F:AAAGCACCCAGAATATAATGAG R: ACTTGCTGTTACCATGTCAAC	(CT)_15_	211	56	JN040551
FgauB130	F:GGAAAAGCCTAGAGAGAGGTG R: CAAAGGGCACATCAGTTAAAG	(GA)_3_GG(GA)_8_	176	56	JN040552

### Detecting SSR alleles

PCR products were separated on high-resolution agarose gel (4% Agarose Tiny HT, Genaxxon Bioscience; dissolved in 1×  TAE buffer, A&A Biotechnology). O’RangeRuler 20 bp DNA Ladder (Thermo Scientific) and Marker DNA M1 (26–501 bp; MR11)[DNA-GDANSK] were used as a size standard. Electrophoresis was prepared using a Sub20 Maxi Submarine Gel instrument (Hoefer), in 1×TAE buffer for two hours. Following this, fluorescent gel imaging was performed using a Syngen Imagine System and Phoretix 1D_software. Images were analysed using Gel Analyzer 2010a.

For tetraploid plants, allelic dosage was estimated according to Truong et al. (2005). The allelic dosage was resolved by estimating the number of alleles in a band according to its peak relative intensity.

### Data analysis

The results obtained for the diploid populations were analysed in FSTAT 2.9.3.2 (Goudet, 2002), GeneAlEx 6.5 (Peakall & Smouse, 2012), Gene Pop on the Web (Raymond & Rousset, 1995; Rousset, 2008), SPAGeDi 1.5 (Hardy & Vekemans, 2002) and Microsoft Excel 2016 (v16.0). The significance of Fis per locus and for all loci was determined with a permutation-based test using Monte Carlo simulations (Goudet, 2002).

A modified analysis was used for the tetraploid population: the observed heterozygosity was calculated according to Thrall & Young (2000), and expected heterozygosity according to Van Puyvelde, Van Geert & Triest (2010) in Atetra software based on 1,000 Monte Carlo permutations and Fis index; its significance per locus and for all loci (tested with a permutation test; based on 1,000 permutations) was determined using SPAGeDi software.

The next step of the analysis was conducted in the ‘polysat v. 1.7’ R package according to Clark & Jasieniuk (2011). In the case of the allotetraploid population of *F. amethystina* (Clark & Drauch Schreier, 2017), alleles were assigned to isoloci. After importing the tetraploid population SSR data to polysat, a preliminary analysis was conducted; no visible internal structure of the population was found to demonstrate any clusters or any highly dissimilar samples from the rest.

The polysat algorithm was then run to determine allele assignment. The function ‘processDatasetAllo’ tests several parameter combinations across all loci in the dataset. The parameters were used as default: one parameter set optimized for no null alleles and no homoplasy, one optimized for homoplasy, and two optimized for null alleles. It was assumed that two subgenomes (SG) were present, both being diploid, with an R parameter of 500.

The ‘FgauB103’ locus was excluded from further analysis due to the presence of positive correlations between alleles. In addition, the loci FgauA04, FgauA111, FgauB119 and FgauB130 were also excluded from isolocus assignment as they were found to have a significant proportion of homoplasious alleles. Thus, problem loci were discarded and the remainder were kept for further use. The tetraploid dataset was recoded and, after joining with the diploid dataset, was subjected to further analysis including the calculation of genetic distance.

For all studied populations, genetic similarity and variance was calculated based on the Lynch distance, calculated among all pairs of samples (Clark, 2019). The results were classified according to populations and visualized by Principal Coordinated Analysis (PCoA) in polysat. The distance matrix of the Lynch distance was then exported to GenAlEx 6.5 (Peakall & Smouse, 2012). Analysis of molecular variance (AMOVA) was then conducted with 999 permutations, according to population and region (i.e., the Alps vs. the Western Carpathians). Pairwise PhiPT statistics for populations were also calculated with 999 permutations.

## Results

### Flow cytometry

**Figure 1:**
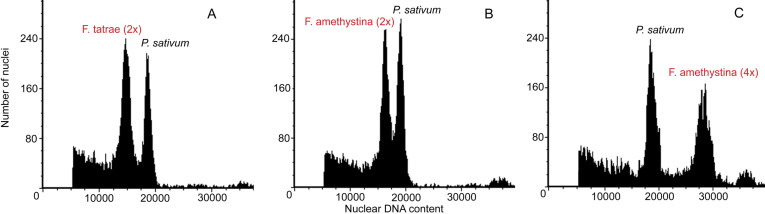
Histograms of nuclear DNA content obtained after FCM analysis of *P. sativum* cv. ‘Set’ (internal standard) and examples of *Festuca* accessions. (A) *F. tatrae* (2×) –Zuberec (Slovakia), (B) *F. amethystina* (2×) –Garmisch-Partenkirchen (Germany) and C) *F. amethystina* (4×) –Mayrwinkl (Austria).

The investigated *F. tatrae* genotypes collected from Zuberec (Slovakia) ranged between 6.83 and 7.38 pg/2C in size (Fig. 1A) indicating that they were diploid. Of the *F. amethystina* accessions, two different 2C DNA content was obtained: those from Garmisch-Partenkirchen (Germany) ranged in size from 7.01 to 7.98 pg/2C (diploid, Fig. 1B), while those collected in Mayrwinkl (Austria), ranged from 12.40 to 14.10 pg/2C (tetraploid, Fig. 1C).

### Identifying accurate SSR markers and genetic distance between complexes

No study has yet been performed of the SSR markers of *F. amethystina* and *F. tatrae*. However, SSR nuclear microsatellite markers designed originally for *Festuca gautieri* were found (Table 1), a plant belonging to the fine-leaved clade within *Festuca* (Segarra-Moragues & Catalán, 2011).

**Figure 2:**
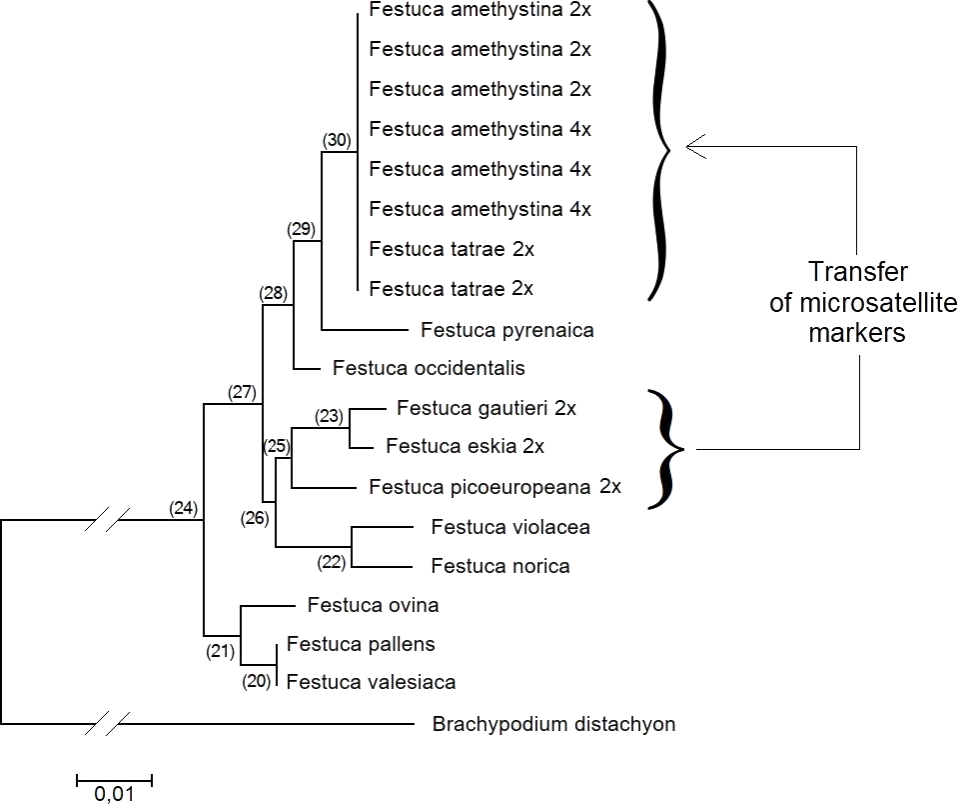
Neighbour-Joining phylogenetic tree based on the analysis of ITS markers of *F. amethystina*—*F. tatrae* complex and related taxa. The evolutionary distances were computed using the Maximum Composite Likelihood method and are given as units of the number of base substitutions per site. In the case of the studied complexes, the level of ploidy of the accessions used in the analysis is shown. The analysis does not include tetraploids from *F. gautieri*.

The analysis of the phylogenetic tree generated from the ITS nuclear marker analysis (Fig. 2) indicated that the genetic distance between the *F. amethystina*–*F. tatrae* complex and the *F. gautieri*–*F. eskia* complex was small, and hence, that SSR markers could be transferred between the two complexes.

### Transferability of protocols

Although the protocols described for *F. gautieri* (Segarra-Moragues & Catalán, 2011) were also effective for *F. amethystina* and *F. tatrae*, the PCR products had weak bands for four markers: FgauA04, FgauA121, FgauB07 and FgauB109. A higher PCR yield was obtained by performing a gradient PCR with modified annealing temperatures and the addition of DMSO (1 µL per 20 µL of mix) (Table 1).

### Characteristics of SSR loci

All 10 microsatellite loci were successfully transferred to *F. amethystina* and *F. tatrae* (Table 2).

**Table 2:** The results of initial primer screening for 10 polymorphic SSR loci in studied populations of *F. amethystina* and *F tatrae*. For each locus, allele range (Ar), number of alleles (Na), observed (Ho) and expected (He) heterozygosities, and inbreeding coefficient (Fis) values are reported for single populations (*N* = 24) of F. amethystina 2×(Garmisch-Partenkirchen, Germany), F. amethystina 4×(Mayrwinkl, Austria) and F. tatrae (Zuberec, Slovakia).^∗^*P* < 0.05,^∗∗^*P* < 0.01;^∗∗∗^*P* < 0.001; ns, not significant (permutation tests; for diploids using Monte Carlo simulations in FSTAT (Goudet, 2002) and tetraploids using the permutation test in SPAGeDi software (Hardy & Vekemans, 2002).

Locus	*F. amethystina* (2×)					*F. amethystina* (4×)					*F. tatrae* (2×)				
	Ar	Na	Ho	He	Fis	Ar	Na	Ho	He	Fis	Ar	Na	Ho	He	Fis
FgauA02	178–338	13	1.000	0.904	−0.086^ns^	146–376	15	0.611	0.898	0.328^***^	178–262	11	1.000	0.865	−0.135^ns^
FgauA04	86–124	3	0.375	0.369	0.005^ns^	92–126	5	0.250	0.483	0.399^*^	86–102	2	0.083	0.497	0.839^**^
FgauA111	216–258	7	0.208	0.847	0.763^**^	230–300	9	0.056	0.854	0.931^***^	216–236	4	0.000	0.604	1^**^
FgauA121	88–160	4	0.500	0.660	0.262^**^	86–162	5	0.334	0.651	0.480^***^	76–164	4	0.500	0.661	0.263^ns^
FgauB07	78–96	3	0.542	0.635	0.167^ns^	92–108	6	0.528	0.786	0.298^**^	94–100	2	0.667	0.444	−0.484^ns^
FgauB103	122–154	8	0.417	0.760	0.469^**^	92–174	8	0.681	0.851	0.222^**^	114–190	11	0.708	0.872	0.208^*^
FgauB109	86–92	2	0.208	0.187	−0.095^ns^	90–98	3	0.167	0.290	0.312^ns^	90–98	2	0.208	0.187	−0.095^ns^
FgauB119	108–152	5	0.875	0.576	−0.502^ns^	80–130	6	0.340	0.795	0.622^***^	106–146	5	0.833	0.734	−0.115^ns^
FgauB125	196–264	12	0.917	0.874	−0.027^ns^	188–282	17	0.722	0.901	0.212^***^	180–290	9	0.625	0.838	0.274^*^
FgauB130	86–182	11	0.542	0.869	0.395^**^	108–118	3	0.083	0.460	0.766^***^	100–124	4	0.125	0.683	0.824^**^
Mean		6.800	0.558 ± 0.090	0.668 ± 0.075	0.185^**^		7.700	0.377 ± 0.233	0,697	0.450^***^		5.400	0.475 ± 0.110	0.638 ± 0.068	0.276^**^

**Notes.**

* *P* < 0.05.

** *P* < 0.01.

*** *P* < 0.001.

ns not significant (permutation tests).

For diploids using Monte Carlo simulations in FSTAT (Goudet, 2002) and tetraploids using the permutation test in SPAGeDi software (Hardy & Vekemans, 2002).

Among the 24 diploid accessions of *F. amethystina* (2×), 68 different SSR alleles were detected from 10 polymorphic microsatellite loci. The number of alleles per locus ranged from two (FgauB109) to 13 (FgauA02) with a mean number of 6.8 (Table 2). Observed heterozygosities ranged from 0.21 (FgauA111 and FgauB109) to about 1.0 (FgauA02). Expected heterozygosities ranged from 0.19 (FgauB109) to 0.91 (FgauA02). Four out of ten loci showed significant heterozygote deficiency (mean F_*IS*_ = 0.19) (Table 2).

Among the 24 tetraploid accessions of *F. amethystina* (4×), a total of 77 different SSR alleles were detected from 10 polymorphic microsatellite loci. The number of alleles ranged from three (FgauB109 and FgauB130) to 17 (FgauB125), with a mean number of eight alleles per locus (Table 2). Observed heterozygosity ranged from 0.056 (FgauA111) to 0.722 (locus FgauB125) and expected heterozygosity ranged from 0.290 (locus FgauB109) to 0.901 (locus FgauB125). All loci, except FgauB109, showed significant heterozygote deficiency (mean F_*IS*_ = 0.45) (Table 2).

Among the 24 accessions of *F. tatrae* (2×), a total of 54 different SSR alleles were detected from 10 polymorphic microsatellite loci. The number of alleles ranged from two (FgauB109, FgauA04 and FgauB109) to 11 (FgauA02 and FgauB103), with the mean number of alleles per locus being 5.4 (Table 2). Observed heterozygosity ranged from null (FgauA111) to about one (locus FgauA02) and expected heterozygosities ranged from 0.19 (locus FgauB109) to 0.87 (locus FgauA02). Five out of 10 loci showed significant heterozygote deficiency (mean F_*IS*_ = 0.28) (Table 2).

### Populations similarity and genetic variance analysis

**Figure 3:**
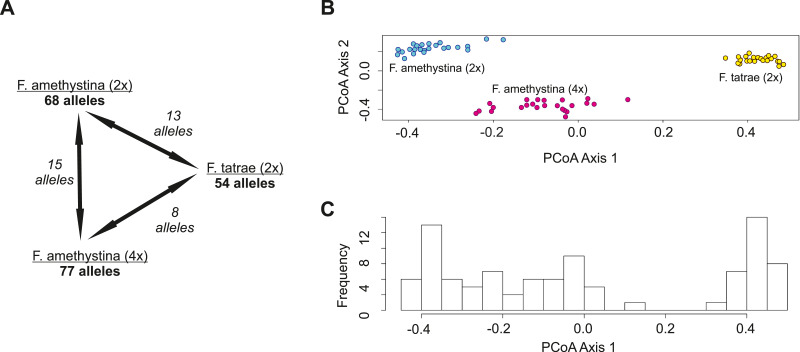
Genetic similarity between studied populations of *F. amethystina* and *F. tatrae* based on 10 SSR polymorphic loci. (A) The total number of alleles and the numbers of alleles shared between populations. (B) Ordination diagram of Principal Coordinates Analysis (PCoA) based on Lynch distance of samples. (C) Frequency of samples along the first PCoA Axis.

In total, 163 different alleles were scored from the 10 SSR loci. No alleles were shared among all studied populations. Only eight alleles were shared between diploid *F. tatrae* (2×) and tetraploid *F. amethystina* (4×) populations (Fig. 3A). The diploid *F. amethystina* population shared 15 alleles with a tetraploid population of *F. amethystina* and 13 alleles with the *F. tatrae* population (Fig. 3A). *F. amethystina* 2×demonstrated 40 exclusive alleles, *F. amethystina* 4×demonstrated 54 alleles and *F. tatrae* 33 alleles.

Principal Coordinates Analysis (PCoA) based on the obtained Lynch distance of samples (accessions) found the samples to form well-separated groups corresponding to species and cytotypes (Fig. 3B). However, *F. amethystina* populations were found to overlap according to the first PCoA axis (Figs. 3B, 3C).

The diploid populations of *F. amethystina* and *F. tatrae* demonstrated similar genetic diversity indices, as measured by observed and expected heterozygosities and inbreeding coefficients*.* However, the tetraploid *F. amethystina* (4×) populations demonstrated higher inbreeding coefficients (Table 2).

Analysis of molecular variance (AMOVA) indicates that a considerable part of the total variance is explained by differentiation within populations (above 60%), one third by differentiation among populations (cytotypes) and only a negligible part is explained by the differences between regions (Alps vs. Carpathians) (Table 3). A close similarity was found between diploid and tetraploid populations of *F. amethystina* (PhiPT = 0.337), and an intermediate similarity between the tetraploid population of *F. amethystina* and population of *F. tatrae* (PhiPT = 0.364); the highest dissimilarity was observed between the diploid population of *F. amethystina* and population of *F. tatrae* (PhiPT = 0.411). All PhiPT values are significant, *p* = 0.01, according to the 999 permutations test.

**Table 3:** Summary of the AMOVA analysis of studied populations: two populations of *F. amethystina* (2×, 4×) and one population of *F. tatrae* (2×). AMOVA was calculated for all pairs of accessions based on the Lynch distance; 999 permutations were used, and both populations and regions (the Alps vs. the Western Carpathians) were taken into account.

Source	df	SS	MS	Est. Var.	%
Among Regions	1	4.516	4.516	0.008	2%
Among Populations	1	4.244	4.244	0.165	36%
Within Populations	69	19.865	0.288	0.288	62%
*Total*	*71*	*28.625*		*0.461*	*100%*

## Discussion

The genome size of the investigated *F. tatrae* genotypes is similar to those of the accessions of *F. tatrae* from Slovakia (2*n* = 2*x* = 7.00 pg/2C) reported by Šmarda et al. (2008). The *F. amethystina* genome sizes, however, suggest that the samples are diploid and tetraploid, which is in line with our previous work (Rewicz et al., 2018).

Following a literature review and phylogenetic analysis we decided to examine the possibility of transferring the microsatellite loci from the Iberian fescue *Festuca gautieri* homoploid hybrid complex to the Central European *F. amethystina*—*F. tatrae* polyploid hybrid complex. There was a high probability of success, as SSR markers often display strong transferability across species within a genus (Gaitán-Solís et al., 2002; Saha et al., 2004).

The species selected for the source of SSR marker transfer, *Festuca gautieri* (2×  and 4×, De la Fuente & Ortúñez, 2001), is a good example of a fine-leaved species possessing a set of easily available SSR markers (Segarra-Moragues & Catalán, 2011). Ten polymorphic microsatellite loci developed for *F. gautieri* had previously been transferred to the closely-related *F. eskia* (2×) and to the interspecies diploid hybrid *F. ×picoeuropeana*. This diploid hybrid complex has been extensively studied based on homoploid hybridization with the aim of unravelling the outcomes of speciation (Marques et al., 2016). It has been subjected to extensive nuclear genotypic analysis by SSR markers with a good degree of success, although the tetraploid form of *F. gautieri* was not included in the analyses.

However, all complex members, including polyploids, were used in the present study (*F. amethystina*–*F. tatrae* complex). The results obtained for *F. amethystina* and *F. tatrae* are generally similar to those achieved by Segarra-Moragues & Catalán (2011) for the *F. eskia* complex, but with different minimum numbers of alleles: the present study identified a smaller mean number of alleles per locus, and while the maximum number of alleles was the same (7), the numbers differed between individual loci. In addition, the observed and expected heterozygosities were found to have wider ranges and lower values.

In our study no alleles were shared among all studied populations. However, some were shared between pairs of populations: eight common alleles between *F. tatrae* and tetraploid population of *F. amethystina*; 15 between a diploid and tetraploid population of *F. amethystina*, and 13 between *F. tatrae* and diploid population of *F. amethystina*. In the *F. gautieri* homoploid complex. Segarra-Moragues & Catalán (2011) found 58 alleles out of 137 to be shared; however, while the populations of *F. amethystina* and *F. tatrae* presented in our present work were separated geographically, their study took samples from more sympatric localities.

Our work serves as an example of how to implement multivariate analyses and AMOVA using microsatellite data in an allopolyploid complex, with Lynch distance being used as the measure of genetic distance. It is recommended that Lynch distance be used for allopolyploids (Clark, 2019). In this measure it is assumed that only one copy of each allele is present, and that two alleles from two individuals are either identical or not; however, the alleles are still grouped by locus, and distances are averaged across all loci (Clark, 2019). The results of PCoA analysis obtained by Lynch distance revealed relationships between diploid and tetraploid populations; in addition, AMOVA showed a partition of variation between SSRs. However, our findings are based only on three example populations, and a deeper analysis is needed using studies based on a higher number of populations selected for appropriate research hypotheses. For example, PCoA analysis has previously demonstrated great potential in the analysis of the described SSR markers in studies on hybridization and introgression in the case of the *F. gautieri*—*F. eskia* complex (Marques et al., 2016); they were also found to identify well-separated populations in the present study.

The availability of SSR markers enables wider research to be performed on species adaptation, e.g., for identifying alleles associated with functional traits (Sun et al., 2015). The *F. amethystina*—*F. tatrae* complex is also a good model for research on that the local adaptability of grasses with different ploidy levels.

Our example of these Iberian fescues demonstrate that microsatellite loci can be likewise used to investigate landscape genetics both across a wide distribution and at a narrower geographical scale. They can also be used as a genetic tool to establish conservation strategies for endangered species.

## Conclusions

The set of transferred SSR markers can be useful in research on the allopolyploid complex of *F. amethystina* (2x and 4x) and *F. tatrae* (2×). All markers were polymorphic, and thus have potential value in studying population genetics and the inheritance relationships within and between cytotypes and species. Transferred markers can be used in research on inbreeding depression in the studied species and on the variation in the degree of genetic diversity between populations of different cytotypes in mountain and lowland areas. These analysis also be employed as components of conservation strategies concerning biodiversity at the genetic level.

##  Supplemental Information

10.7717/peerj.9227/supp-1 Data S1 The raw measurements of microsatellite for Festuca amethystina 2× The columns in the table are described by SSR marker data, while the rows represent specimens (in total: 10 SSR markers and 24 specimens).

10.7717/peerj.9227/supp-2 Data S2 The raw measurements of microsatellite for Festuca amethystina 4× The columns in the table are described by SSR marker data, while the rows represent specimens (in total: 10 SSR markers and 24 specimens).

10.7717/peerj.9227/supp-3 Data S3 The raw measurements of microsatellite for Festuca tatrae The columns in the table are described by SSR marker data, while the rows represent specimens (in total: 10 SSR markers and 24 specimens). Columns are semicolon-delimited.

10.7717/peerj.9227/supp-4 Data S4 DNA sequences of ITS region from F. amethystina and Festuca tatrae Sequences of ITS marker from *F. amethystina* 2×(3 sequences, label: FAME_ITS_AM), *Festuca amethystina* 4×(3 sequences, label: FAME_ITS_GP) and from F. tatrae 2×(2 sequences, label: FTAT_ITS_PP). Sequences are available at GenBank: MN783289 –MN783296.

## Additional Information and Declarations

### Field Study Permissions

The following information was supplied relating to field study approvals (i.e., approving body and any reference numbers): Studied species are not protected by law in Slovakia (Zákon č. 543/2002 Z. z. O Ochrane Prírody a Krajiny) or Lands in Germany and Austria (Bayern, confirmation from Botanische Staatssammlung München; Upper Austria, confirmation from Direktion für Landesplanung, wirtschaftliche und ländliche Entwicklung Abteilung Naturschutz) where there were taken. Samples (a few leaves from each specimen) were collected outside protected areas.

### DNA Deposition

The following information was supplied regarding the deposition of DNA sequences: Sequences are available in the Supplemental Files and at GenBank: MN783289, MN783290, MN783291, MN783292, MN783293, MN783294, MN783295, MN783296.

### Data Availability

The following information was supplied regarding data availability: The raw measurements of microsatellite are available in the Data S1–S3).
